# A Novel Detection Scheme in Image Domain for Multichannel Circular SAR Ground-Moving-Target Indication

**DOI:** 10.3390/s22072596

**Published:** 2022-03-28

**Authors:** Qinghai Dong, Bingnan Wang, Maosheng Xiang, Zhongbin Wang, Yachao Wang, Chong Song

**Affiliations:** 1 National Key Laboratory of Microwave Imaging Technology, Aerospace Information Research Institute, Chinese Academy of Sciences, Beijing 100094, China; dongqh@aircas.ac.cn (Q.D.); xms@mail.ie.ac.cn (M.X.); wangzhongbin17@mails.ucas.ac.cn (Z.W.); wangyc@aircas.ac.cn (Y.W.); songchong18@mails.ucas.edu.cn (C.S.); 2 School of Electronic, Electrical and Communication Engineering, University of Chinese Academy of Sciences, Beijing 100094, China

**Keywords:** multichannel circular SAR, ground-moving-target indication (GMTI), clutter suppression, along-track interferometry (ATI), the heterogeneous clutter environment

## Abstract

Circular synthetic aperture radar (CSAR), which can observe the region of interest for a long time and from multiple angles, offers the opportunity for moving-target detection (MTD). However, traditional MTD methods cannot effectively solve the problem of high probability of false alarm (PFA) caused by strong clutter. To mitigate this, a novel, three-step scheme combining clutter background extraction, multichannel clutter suppression, and the degree of linear consistency of radial velocity interferometric phase (DLRVP) test is proposed. In the first step, the spatial similarity of the scatterers and the correlation between sub-aperture images are fused to extract the strong clutter mask prior to clutter suppression. In the second step, using the data remaining after elimination of the background clutter in Step 1, an amplitude-based detector with higher processing gain is utilized to detect potential moving targets. In the third step, a novel test model based on DLRVP is proposed to further reduce the PFA caused by isolated strong scatterers. After the above processing, almost all false alarms are excluded. Measured data verified that the PFA of the proposed method is only 20% that of the comparison method, with improved detection of slow and weakly moving targets and with better robustness.

## 1. Introduction

Synthetic aperture radar (SAR) is widely used in military and civil fields due to its high-resolution imaging of ground scenes, large coverage area, and all-weather capability [1,2,3]. The ground-moving-target detection mode of SAR is playing an increasingly important role for battlefield surveillance, traffic monitoring, and other applications [4,5,6,7].

The commonly used moving-target detection methods can be divided into two types: single-channel [5,8,9,10] and multichannel [3,11,12,13,14]. The single-channel moving-target-detection method uses the Doppler shift and Doppler rate characteristics of moving targets in SAR images [5,9]. The Doppler-shift method based on a Doppler frequency filter can only detect targets whose Doppler is outside the clutter spectrum (the clutter spectrum is the Doppler spectrum [15] formed by radar echoes generated by uninteresting reflectors), so it is not suitable for detecting targets with slow radial velocity. On the other hand, the method based on Doppler-rate estimation requires iteration or time-frequency analysis, which is computationally expensive. In order to improve the performance of parameter estimation and reduce the computational complexity of single-channel GMTI, Wang [4] proposed a method for moving-target trajectory reconstruction in single-channel CSAR by using prior knowledge of road information. In the Radon–Wigner distribution domain, an efficient estimation method using geometry information is proposed to realize the calculation of two parameters instead of extensive parameter search; this effectively reduces computational complexity [10]. In [16], a fast ground-moving-target indication and parameter estimation algorithm based on prior knowledge of road networks is proposed, which effectively reduces the calculation required. However, it is often difficult to obtain road information in real-time, especially for military applications. Due to the lack of spatial degrees-of-freedom in azimuth, the single channel system cannot perform clutter suppression. Thus, it is difficult to detect slowly moving targets and low signal-to-clutter-ratio (SCR) targets. The above disadvantages tremendously restrict the application of the single-channel system.

Different from the single-channel system, the multichannel system has more spatial degrees-of-freedom in azimuth and can realize clutter suppression, thus improving SCR, which effectively enhances detection and parameter estimation performance for slowly moving targets [17]. To detect slowly moving targets submerged in the main-lobe clutter spectrum, multichannel methods such as displaced phase center antenna (DPCA) [11,18,19], ATI [7,20,21], space–time adaptive processing (STAP) [12,22], and robust principal component analysis (RPCA) [23,24,25] have been widely developed. DPCA and ATI are sensitive to the inevitable noise and are suboptimal for systems with more than two channels. STAP can reduce the minimum detectable velocity in a strong clutter environment by keeping the energy of the moving target constant while minimizing the output energy of clutter [12,26]. Unfortunately, the computational complexity of STAP is high due to having to search the maximum of the test statistic and calculate the clutter covariance matrix pixel by pixel. In addition, the calculation of the clutter covariance matrix requires a large number of homogeneous clutter training samples without moving targets, which is difficult to meet in a heterogeneous environment [26]. By the fact that the moving targets are spatially sparse and have linear interferometric phases between different channels, RPCA can distinguished strong stationary clutters with low rank structure [23,24,25].

However, when large radar cross section (RCS) or highly heterogeneous clutter exist, the residual energy of interference is still strong after using the above clutter suppression methods, which will lead to a significant number of false alarms and reduce the accuracy of parameter estimation [27,28]. The so-called strong clutter residues in this paper are mostly produced by common artificial scatterers. These objects have a large RCS. Limited by the ability of clutter suppression, there will still be strong residual energy after clutter suppression. The existing methods are difficult to adapt to the complex environment, especially in urban areas.

Based on the above analysis, it can be concluded that if we can extract the position distribution of a large RCS from a SAR image in advance, clutter suppression can eliminate the strong clutter. In order to implement this idea, we propose a new method to extract the position of large RCS from sub-aperture images. Different from linear SAR, a CSAR system moves along a circular trajectory, which enables long-time and multi-aspect observation of a scene [4,29,30].

In fact, strong reflectors can be considered isotropic and coherent when the difference in viewing angles is small [5,31,32,33]. In addition, pixels of these static scatterers in the SAR image have the characteristics of large amplitude, continuous distribution, and strong spatial structure. Therefore, we can extract position information of a strong clutter background by using the spatial structure information of strong scatterers and the correlation between multi-angle observations.

In order to realize the above purpose, a novel, three-step scheme based on strong background extraction, multichannel clutter suppression, and the DLRVP test is proposed. In the first step, the spatial similarity of the scatterers themselves and the correlation between the sub-aperture images are fused to extract the strong clutter background as the prior information of large-RCS clutter suppression. To avoid interference of large RCS targets, a double-threshold method is used to extract the clutter background. In the second step, for the data after clutter suppression and strong clutter background elimination, a new, amplitude-based detector is utilized to detect potential moving targets with a relatively low threshold. After the above two steps, most of the false-alarm targets have been excluded, and the position of each potential moving target in the image is obtained. However, there are still some isolated strong scattering points or edges of man-made objects that cannot be eliminated by the above two steps. In the third step, a novel detection model based on the linear consistency of the interference phase is constructed to reduce false alarm targets caused by isolated strong clutter.

The flow of the proposed method is summarized as follows. Step I: Extract the coordinates of strong static clutter in the observed scene; use ‘1’ to indicate that the pixel is strong clutter and ‘0’ to indicate that the pixel is non-strong clutter, thus forming a background-mask map. Step II: Register the image to be detected with the mask map generated by Step I. Remove the pixels in the strong clutter position, and the remaining pixels are used for target detection. Step III: Calculate the DLRVP value of each target detected by Step II, and perform CFAR detection again to eliminate false alarm targets in the detection results of Step II.

In this paper, four-channel, airborne, X-band data is used to verify the correctness and effectiveness of the proposed method. We compared the detection performance of the proposed method with the DRVC method [14], the GO-DPCA method [11], and the weighted DPCA [34]. The results of measured data verified that the PFA of the proposed method is only 20% that of the comparison method and has better robustness.

The reminder of this paper is structured as following. Section 2 introduces the system geometry and the signal model of CSAR GMTI. In Section 3, the proposed three-step scheme is described in detail and compared with existing detection methods through simulation analysis. In Section 4, the effectiveness of the proposed method is demonstrated by using actual data collected by a four-channel airborne CSAR system. Finally, according to the results of simulated and measured data, we summarize the proposed method in Section 5.

## 2. Geometry and Signal Model of Multichannel CSAR System

This paper takes the sub-aperture image obtained by the back-projection (BP) algorithm as the research object. In this section, the image geometry and the moving-target model of multichannel CSAR GMTI are described.

### 2.1. Geometry

**Figure 1:**
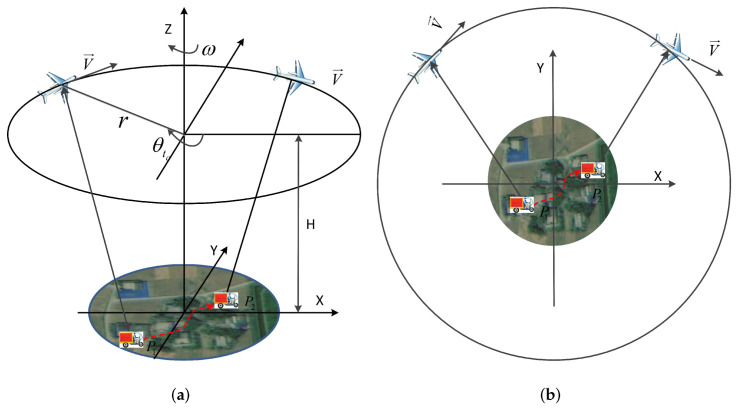
The observation geometry of multichannel CSAR-GMTI. The red dashed line represents the path of a moving target; coordinates $P_{1}$ and $P_{2}$ represent the locations of the same moving target in different sub-aperture images: (**a**) lateral view; (**b**) vertical view.

The observation geometry of a multichannel CSAR-GMTI system is shown in Figure 1. The platform moves at a constant angular velocity ω along a circular track with radius *r* and hovering height *H* and has *M* spatial channels with a fixed distance *d* that are parallel to the flying direction. The first channel serves as both a transmitting and receiving channel, while the other channels only receive echoes. Let $t_{a}$ denote the slow time variable, so the instantaneous azimuth angle of the antenna phase center (APC) of the reference channel can be expressed as $\theta_{t_{a}}=\theta_{0}+\omega t_{a}$, where $\theta_{0}$ is the initial azimuth angle of the reference channel. The instantaneous coordinates $C_{i}={\left(x_{i},y_{i},z_{i}\right)}^{T}$ of the $i_{th}$$\left(i=1,2\cdots M\right)$ APC are given by [30]:

(1) $$\begin{array}{c} x_{i}\left(t_{a}\right)=r\cos\left(\theta_{t_{a}}\right)+\left(i-1\right)d\sin\left(\theta_{t_{a}}\right)\\ y_{i}\left(t_{a}\right)=r\sin\left(\theta_{t_{a}}\right)-\left(i-1\right)d\cos\left(\theta_{t_{a}}\right)\\ z_{i}\left(t_{a}\right)=H \end{array}.$$

At time $t_{a}=0$, a ground-moving target is located at position $P_{0}={\left(x_{0},y_{0},z_{0}\right)}^{T}$. The moving target is assumed to move along a straight line at a constant velocity on flat ground. The velocities of the moving target along the X-Y-Z axis are $v_{x}$, $v_{y}$, $v_{z}$, respectively. Thus, the instantaneous coordinates $P={\left(x_{p},y_{p},z_{p}\right)}^{T}$ of the moving target can be written as following [30]:

(2) $$\begin{array}{c} x_{p}\left(t_{a}\right)=x_{0}+v_{x}t_{a}\\ y_{p}\left(t_{a}\right)=y_{0}+v_{y}t_{a}\\ z_{p\left(t_{a}\right)}\;=z_{0}=h \end{array}$$

where *h* denotes the altitude of the observation scene.

Based on the above analysis, we can deduce that the instantaneous slant range $R_{i}\left(t_{a}\right)$ between the APC of the $i_{th}$ channel and the moving target can be expressed as [29,30]:

(3) $$\begin{array}{c} R_{i}\left(t_{a}\right)=\left|\vec{OP}-\vec{OC_{i}}\right|\;\\ \;\;\;=\sqrt{{\left(x_{i}\left(t_{a}\right)-x_{p}\left(t_{a}\right)\right)}^{2}+{\left(y_{i}\left(t_{a}\right)-y_{p}\left(t_{a}\right)\right)}^{2}+{\left(H-h\right)}^{2}} \end{array}$$

where $v_{ta}$ and $v_{tr}$ are defined as the projection of target’s motion velocity in the flight direction and the radial direction of the platform at time $t_{a}$, respectively.

For the $k_{th}$ sub-aperture, the second-order Taylor series expansion of (3) at $t_{a}=t_{k}$ can be written:

(4) $$R_{i}\left(t_{a}\right)\approx R_{i}\left(t_{k}\right)+{R_{i}}^{\prime }\left(t_{k}\right)\left(t_{a}-t_{k}\right)+{R_{i}}^{\prime \prime }\left(t_{k}\right){\left(t_{a}-t_{k}\right)}^{2}.$$

Let $x_{p,t_{k}}$ and $y_{p,t_{k}}$ represent the position coordinates of the target at $t_{k}$. So the primary and secondary Taylor expansion coefficients can be derived as follows:

(5) $$\begin{array}{c} R_{i}^{{}^{\prime }}\left(t_{k}\right)\\ =\frac{r\omega \left(y_{p,t_{k}}\cos\left(\theta_{t_{k}}\right)-x_{p,t_{k}}\sin\left(\theta_{t_{k}}\right)\right)}{R_{i}\left(t_{k}\right)}+\frac{\left(i-1\right)d\left(v_{ta}-\omega \left(x_{p,t_{k}}\cos\left(\theta_{t_{k}}\right)-y_{p,t_{k}}\sin\left(\theta_{t_{k}}\right)\right)\right)}{R_{i}\left(t_{k}\right)}+v_{tr} \end{array}$$

(6) $${R_{i}}^{\prime \prime }\left(t_{k}\right)=\frac{v_{x}^{2}+v_{y}^{2}-2v_{ta}r\omega +\omega^{2}r\left(x_{p,t_{k}}\cos\left(\theta_{t_{k}}\right)+y_{p,t_{k}}\sin\left(\theta_{t_{k}}\right)\right)-R_{i}{{}^{\prime }}^{2}\left(t_{k}\right)}{2R_{i}\left(t_{k}\right)}$$

where

(7) $$v_{ta}=-v_{x}\sin\left(\theta_{t_{k}}\right)+v_{y}\cos\left(\theta_{t_{k}}\right)$$

(8) $$v_{tr}=\frac{v_{x}x_{p,t_{k}}+v_{y}y_{y,t_{k}}-r\left(v_{x}\cos\left(\theta_{t_{k}}\right)+v_{y}\sin\left(\theta_{t_{k}}\right)\right)}{R_{i}\left(t_{k}\right)}$$

### 2.2. Signal Model

**Figure 2:**
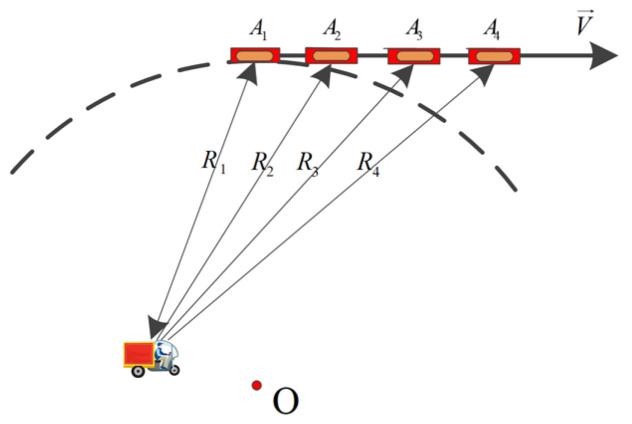
The geometry of ATI for CSAR (the red dot represents the center of the observation scene).

The adjacent antennas are distributed along the circular flight track in Figure 2. The system adopts a single-channel transmitting and all-channel receiving working mode. Regard Channel 1 as the reference channel and the other channels as the auxiliary channels. The chirp signal is used as the transmitting signal. In this mode, the echo of the auxiliary antenna has a bistatic effect. After range compression, the echo signals in the $k_{th}$ sub-aperture received by the reference channel and the $i_{th}$ channel can be written, respectively, as [11,35]:

(9) $$S_{1}\left(t_{r},t_{a}\right)=\sigma_{0}rect\left[\frac{t_{a}-t_{k}}{T_{sub}}\right]\mathrm{sinc}\left\{\pi B\left(t_{r}-\frac{4R_{1}\left(t_{a}\right)}{c}\right)\right\}exp\left[-j\frac{2\pi f_{c}R_{1}\left(t_{a}\right)}{c}\right]$$

(10) $$\begin{array}{c} S_{i}\left(t_{r},t_{a}\right)\\ =\sigma_{0}rect\left[\frac{t_{a}-t_{k}}{T_{sub}}\right]\mathrm{sinc}\left\{\pi B\left(t_{r}-\frac{R_{1}\left(t_{a}\right)+R_{i}\left(t_{a}\right)}{c}\right)\right\}exp\left[-j\frac{2\pi f_{c}}{c}\left(R_{1}\left(t_{a}\right)+R_{i}\left(t_{a}\right)\right)\right] \end{array}$$

where $\sigma_{0}$ is represents the amplitude of the echo, $t_{r}$ is fast time, and *c* is the speed of light. The symbol *B* is frequency bandwidth, $f_{c}$ is carrier frequency, rect(·) is the rectangle window, and $T_{\mathrm{sub}}$ and $t_{k}$ denote the synthetic aperture time and center time, respectively, of the $k_{th}$ sub-aperture.

According to the principle of equivalent phase center [35,36,37], after compensating for the constant phase term, the phase centers of the auxiliary channel can be considered equivalent to a single transmitting and receiving antenna. After co-registration with respect to the reference channel in the azimuth direction, the slant distance between the target and the $i_{th}$ antenna can be written [35]:

(11) $$2R_{i,reg}\left(t_{a}\right)=R_{1}\left(t_{a}+\Delta t_{i,a}\right)+R_{i}\left(t_{a}+\Delta t_{i,a}\right)$$

where $\Delta t_{a}$ is the time delay caused by the azimuth displacement between the phase center of the $i_{th}$ channel and reference channel; it can be calculated as follow:

(12) $$\tan\left(\omega \Delta t_{i,a}\right)=\frac{-\left(i-1\right)d}{2r}\approx \omega \Delta t_{i,a}.$$

For the $i_{th}$ channel, there exists $d_{i}\left(t_{a}\right)$ that satisfies Equation (13):

(13) $$\begin{array}{c} 2R_{i,reg}\left(t_{a}\right)=R_{1}\left(t_{a}+\Delta t_{i,a}\right)+R_{i}\left(t_{a}+\Delta t_{i,a}\right)\\ \;\;\;\;=2R_{1}\left(t_{a}\right)+d_{i}\left(t_{a}\right) \end{array}.$$

Substituting Equations (12) and (13) into Equation (4), we can obtain:

(14) $$\begin{array}{c} d_{i}\left(t_{a}\right)=R_{1}\left(t_{a}+\Delta t_{i,a}\right)+R_{i}\left(t_{a}+\Delta t_{i,a}\right)-2R_{1}\left(t_{a}\right)\\ \;\;=R_{i}\left(t_{k}\right)-R_{1}\left(t_{k}\right)+2{R_{1}}^{\prime }\left(t_{k}\right)\Delta t_{i,a}+\left({R_{i}}^{\prime }\left(t_{k}\right)-{R_{1}}^{\prime }\left(t_{k}\right)\right)\left(t_{a}+\Delta t_{i,a}-t_{k}\right)\\ \;\;+\left({R_{i}}^{\prime \prime }\left(t_{k}\right)-{R_{1}}^{\prime \prime }\left(t_{k}\right)\right){\left(t_{a}+\Delta t_{i,a}-t_{k}\right)}^{2}+2{R_{1}}^{\prime \prime }\left(t_{k}\right)\left[2\Delta t_{i,a}\left(t_{a}-t_{k}\right)+\Delta t_{i,\alpha }^{2}\right]\\ \;\;=v_{tr}\Delta t_{i,a}+R_{n}\left(t_{a}\right) \end{array}$$

where $R_{n}\left(t_{a}\right)$ is a polynomial about $t_{a}$. In the synthetic aperture time, the value of $R_{n}\left(t_{a}\right)$ is less than λλ1616, so it can be ignored.

After delay correction, the signal of channel $i_{th}$ can be expressed as [3,30]:

(15) $$S_{i}\left(t_{r},t_{a}\right)=\sigma_{0}rect\left[\frac{t_{a}-t_{k}}{T_{sub}}\right]\sin c\left\{\pi B\left(t_{r}\;-\;\frac{2R_{i,reg}\left(t_{a}\right)}{c}\right)\right\}exp\left[-j\frac{4\pi f_{c}}{c}R_{i,reg}\left(t_{a}\right)\right].$$

Based on the above discussion, after azimuth-matched filtering, the interferometric phase of the moving target between the $i_{th}$ channel and the reference channel can be calculated by [3,19,30]:

(16) $$\varphi_{ATI}=arg\left(Z_{1}Z_{i}^{*}\right)=\frac{2\pi d_{i}\left(t_{a}\right)}{\lambda }\approx -\frac{2\pi \left(i-1\right)dv_{tr}}{\lambda r\omega }$$

where $Z_{1}$ and $Z_{i}$ are complex images of the first and $i_{th}$ channels, respectively, arg() returns the phase of a complex number, ${\left[\cdot \right]}^{*}$ is the sign of complex conjugate, and λ is the wavelength of the radiation.

From (16), we can get the spatial steering vectors of a moving target and the stationary clutter in the image domain, which can be written as [3]:

(17) $$\begin{array}{c} \alpha_{s}\left(v_{tr}\right)={\left[1,e^{j\frac{2\pi dv_{tr}}{\lambda r\omega }},\cdots ,e^{j\frac{2\pi \left(M-1\right)dv_{tr}}{\lambda r\omega }}\right]}^{T}\in C^{M\times 1}\\ \alpha_{C}={\left[1,1,\cdots ,1\right]}^{T}\in C^{M\times 1} \end{array}$$

where ${\left[\cdot \right]}^{T}$ denotes the transpose of the matrix.

After time-delay compensation between channels, SAR focusing, image registration, and phase calibration to make each pixel independent and identically distributed, the complex data of pixel $\left(p,q\right)$ in M images can be arranged in a vector $Z\left(p,q\right)$ by the following model [3,38,39]. Two statistical hypotheses have to be distinguished:

(18) $$\begin{array}{c} H_{0}:Z\left(p,q\right)=Z_{C}+Z_{W}=\delta_{C}\left(p,q\right)\alpha_{C}+n\left(p,q\right)\\ H_{1}:Z\left(p,q\right)=Z_{T}+Z_{C}+Z_{W}=\delta_{S}\left(p,q\right)\alpha_{S}\left(v_{tr}\right)+\delta_{C}\left(p,q\right)\alpha_{C}+n\left(p,q\right) \end{array}$$

where $Z\left(p,q\right)$, $\delta_{C}\left(p,q\right)$, $\delta_{s}\left(p,q\right)$, $n\left(p,q\right)$ represent the complex data of the processed SAR, the stationary ground clutter, the moving target, and the white noise signal, respectively. The clutter vector $Z_{C}$ and white vector $Z_{W}$ are assumed to be circularly symmetric, complex, Gaussian, random vectors, that is, mutually uncorrelated real and imaginary parts, with zero means. The samples of $Z_{C}$ are highly mutually correlated because they are obtained from the same perspective in short time interval. Hypothesis $H_{0}$ means that a moving target is absent, and hypothesis $H_{1}$ assumes that a moving target exists. Therefore, we can detect the slowly moving target submerged in the main lobe of the clutter spectrum according to the difference of steering vectors between moving target and static clutter.

## 3. The Proposed Detection Scheme for CSAR GMTI

The main goal of this chapter is to develop a new detection method for slowly moving targets that can effectively reduce the false alarm rate caused by strong clutter. For uninteresting targets, we use two methods to remove the impact on detection. First, if the Doppler frequency of the irrelevant target is in the side-lobe of the clutter spectrum, we can remove it by bandpass filtering in the Doppler domain. Second, if the Doppler frequency of the irrelevant target is in the main lobe of the clutter spectrum, we can remove it during tracking, localization, and target identification.

**Figure 3:**
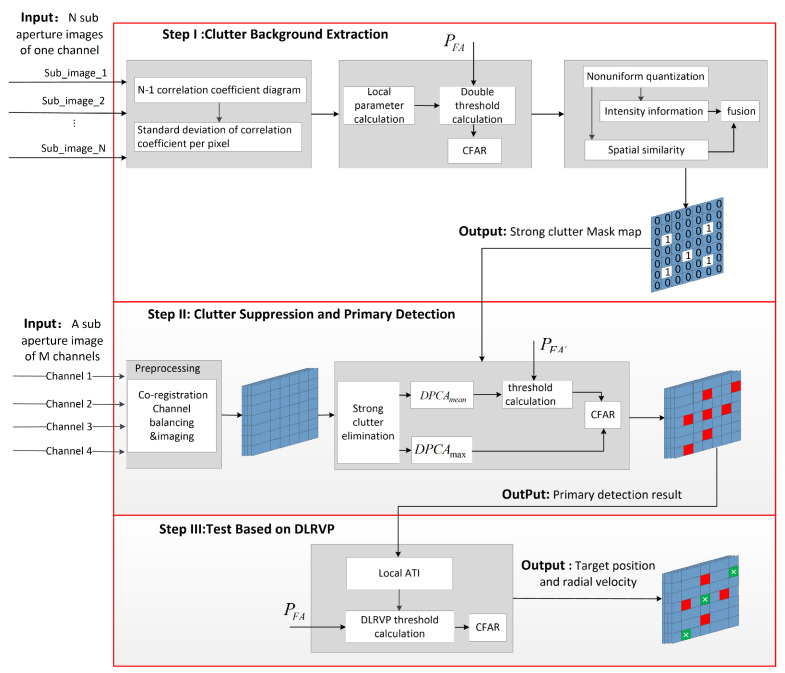
The flowchart of the proposed detection scheme. Each square contains multiple pixels: the ‘1’ square denotes strong clutter, the red square denotes the position of the moving targets detected, and the green square denotes the position of false alarms.

The proposed detection scheme mainly includes three steps, and its detailed flow chart is shown in Figure 3. Firstly, multi-angle sub-aperture imaging obtained by the circular SAR is used to extract the strong clutter background as the prior information for the next step. Secondly, for the multichannel sub-aperture image after image registration, channel equalization, and interferometric phase correction, the method proposed in Section 3.2 is used for clutter suppression and primary detection. In this step, in order to improve the probability of detection and further reduce detection omissions, we used a lower detection threshold when performing CFAR. At this point, the location of the potential target is obtained. Finally, for the potential moving targets obtained in the previous step, the new detection method proposed in Section 3.3 is used for secondary detection to reduce false alarms caused by isolated strong scattering points.

In Section 3.1, Section 3.2 and Section 3.3, the three key components of the detection method proposed in this paper will be analyzed in detail.

### 3.1. Step I: Clutter Background Extraction

Without prior information, it is difficult to eliminate the false alarm caused by strong background clutter. In fact, strong scatterers are mostly artificial targets such as houses, vehicles, and bridges. When the observation angle difference is small, they can be considered as isotropic and coherent. In addition, the pixels of these static scatterers in the SAR image have the characteristics of large amplitude, continuous distribution, and strong spatial structure. Therefore, we can extract position information of the strong clutter background by using the spatial structure of strong scatterers and the correlation between multi-angle observations.

This section designs a method to extract strong clutter background by combining sub-aperture correlation, clutter signal strength, and spatial similarity information. Without changing the basic distribution of actual clutter, the contrast information between strong clutter and surrounding clutter is maximized so as to accurately extract the position information of strong scatterers in the scene.

The process of extracting the strong clutter background is mainly divided into two steps. Firstly, we divide 360 degrees into N sub-apertures equally [40] (the size of the sub-aperture is determined by the SAR resolution [4,8]), and the two adjacent sub-apertures partially overlap. Then, the radar echoes received by each sub-aperture are imaged to obtain N sub-aperture images. Calculate the correlation coefficients of two adjacent sub-aperture images to obtain N-1 correlation coefficient maps. Then the appropriate threshold is selected according to the statistical histogram of the correlation coefficients of all sub-apertures. For a single pixel location, when the corresponding N-1 correlation coefficients are all greater than the threshold, the pixel location is considered to be a strong scatterer. Secondly, the strong clutter detected in the previous step and its surrounding pixels are selected for amplitude and spatial similarity detection, so as to extract the structural information of the entire strong scatterer. The strong clutter background obtained in the above two steps is used as the prior information for later detection to reduce the probability of false alarms and improve the detectability of weak targets.

This article only cares about the amplitude of the correlation coefficient, not its phase. With reference to the classic calculation method of sample cross-correlation coefficient [38], the correlation coefficient between adjacent sub-aperture images can be written as:

(19) $$\begin{array}{c} \gamma =\left|\frac{\sum_{k=1}^{K}C_{1}\left(k\right)C_{2}^{*}\left(k\right)}{\sqrt{\sum_{k=1}^{K}{\left|C_{1}\left(k\right)\right|}^{2}}\sqrt{\sum_{k=1}^{K}{\left|C_{2}\left(k\right)\right|}^{2}}}\right|,0⩽\gamma ⩽1 \end{array}$$

where $C_{1}\left(k\right)$ and $C_{2}\left(k\right)$ represent the pixel values of two adjacent sub-apertures, * denotes complex conjugate, and *K* is the number of pixels included in the sample window.

The bilateral CFAR detection method is used to jointly detect the amplitude and spatial similarity of the strongly correlated pixels and their surrounding pixels, so as to extract the continuous, concentrated, and strong structure information of the strong scatterers. Its mathematical model can be written as [34,39,41]:

(20) $$\begin{array}{c} I_{combined_{j}}={I_{intensity}}_{j}\cdot {I_{spatial}}_{j} \end{array}$$

where ${I_{intensity}}_{j}$ is the intensity information of each pixel of the image, and ${I_{spatial}}_{j}$ represents the spatial similarity between each pixel in the image and its neighboring pixels, which is determined by the geometry and spatial distribution of the scatterer. The two represent different target features. The purpose of multiplying the two in Equation (20) is to construct a detection method that considers both image intensity information and spatial distribution information.

Assuming that the intensity of the center pixel of the sliding window $N\left(\omega \right)$ is $I_{x,y}$, $I_{j}$ represents the intensity of the surrounding pixels, and $f_{h}$ is the kernel density estimator with the standard normal distribution as the kernel function. Then the similarity between adjacent pixels characterized by the kernel density estimator can be written as [41]:

(21) $$\begin{array}{c} I_{spatial_{j}}=\frac{f_{h=1}\left(I\right)-\min\left(f_{h=1}\left(I\right)\right)}{\max\left(f_{h=1}\left(I\right)\right)-\min\left(f_{h=1}\left(I\right)\right)}\\ f_{h=1}\left(I_{x,y}\right)=\sum_{j\in N\left(\omega \right)}\exp(-\frac{1}{2}{\left(I_{x,y}-I_{j}\right)}^{2}) \end{array}$$

The kernel density estimator is used to characterize the similarity information between pixels. Compared with the detection method that only uses intensity information, it can well reflect the aggregation degree and structure information of the strong scatterers in the image.

After processing by (20), an image whose amplitude and spatial similarity have been fused is obtained, and then CFAR detection is performed on it, thereby extracting the strong clutter background.

As shown in Figure 3, the detailed process of strong clutter background extraction is as follows:

1. Divide the circumference into N sub-apertures, obtain N SAR images, and register the N images.

2. Calculate the correlation coefficient of two adjacent sub-aperture images, obtain N-1 correlation coefficient maps, calculate the mean value and variance of the correlation table coefficient of each pixel point, and generate a correlation coefficient statistical histogram and a variance histogram. Calculate the correlation coefficient threshold $\rho_{th}$ and the correlation coefficient variance threshold $\rho_{std,th}$ and perform CFAR detection.

3. Select the pixel detected in the previous step and its surrounding pixels and calculate the spatial similarity according to Equation (21).

4. Use Equation (20) to perform joint detection of amplitude and spatial similarity to generate a mask map composed of ‘0’ and ‘1’.

### 3.2. Step II: Clutter Suppression and Primary Detection

Take the first channel as the reference channel, and subtract the image of the reference channel from the images of the second to *M*${}_{th}$ channels to obtain M-1 residual images. After clutter suppression, the difficult problem we face is how to use M-1 residual clutter images to achieve high-performance CFAR detection. Traditional methods usually perform CFAR detection on each residual image, such as the subspace projection (SP) [22,42] method and the images differential (ID) [43,44] method, and then synthesize the CFAR results of M-1 times to obtain the final detection results. These methods only use a single baseline when detecting a residual image, which cannot guarantee that moving targets with different radial velocities can obtain the highest processing gain, resulting in missed target detection. In addition, these methods require M-1 times CFAR detection, and there is no doubt that their computational complexity will be very high.

**Figure 4:**
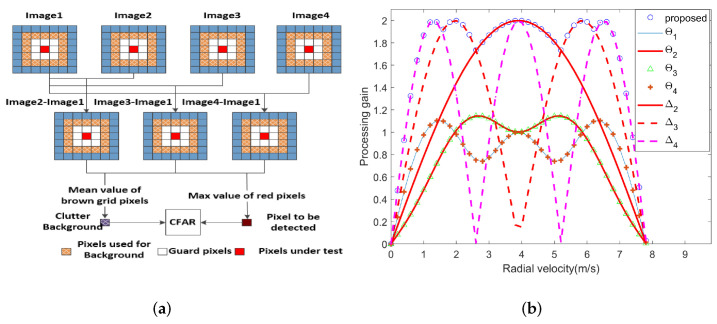
The principle of Step II and gain curves. Θ corresponds to SP algorithm; Δ corresponds to ID algorithm). (**a**) The flowchart of Step II; (**b**) The processing gain curves of moving targets by three methods.

In order to improve the target-detection performance of traditional methods, referring to the idea of GO-CFAR, a CFAR method combined with multichannel DPCA is designed in this paper. The data of pixel $\left(p,q\right)$ in M-1 residual images can be arranged in a vector DPCA$\left(p,q\right)$ by the following model. We take the data with the largest energy in this vector as the detection data, and the mean value of the vector as the background data, so as to obtain two new images. The detailed processing flow of go DPCA is shown in Figure 4a. The pixel to be detected and background pixel value can be expressed as:

(22) $$\begin{array}{c} {\mathrm{DPCA}}_{\max}\left(p,q\right)=\max\left(\left|DPCA_{1}\left(p,q\right)\right|,\cdots ,\left|DPCA_{M-1}\left(p,q\right)\right|\right)\\ {\mathrm{DPCA}}_{\mathrm{mean}}\left(p,q\right)=\mathrm{mean}\left(\left|DPCA_{1}\left(p,q\right)\right|,\cdots ,\left|DPCA_{M-1}\left(p,q\right)\right|\right). \end{array}$$

The two images are jointly used for moving-target detection. The processing gains of three methods are compared and analyzed, as shown in Figure 4b. It can be seen from the simulation results that the processing gain of the SP algorithm is low, and the ID algorithm is prone to speed ambiguity, which leads to missing alarms, while the proposed method has the highest processing gain and a wide speed-adaptation range. In order to prevent the moving-target signal from leaking to the adjacent unit, in addition to the pixels to be detected, several adjacent pixels should be selected as the protection unit. The protection unit does not participate in the estimation of the background clutter, helping to avoid the interference of the moving-target signal on the background.

Next, we will analyze the hypothesis test model and probability distribution characteristics of the proposed method. Amplitude statistical hypotheses of the pixel $\left(p,q\right)$ of the $m_{th}$ channel are given by:

(23) $$\begin{array}{c} H_{0}:\rho \left(p,q\right)=\left|DPCA_{C,m}\left(p,q\right)+DPCA_{N,m}\left(p,q\right)\right|\\ H_{1}:\rho \left(p,q\right)=\left|DPCA_{T,m}\left(p,q\right)+DPCA_{C,m}\left(p,q\right)+DPCA_{N,m}\left(p,q\right)\right| \end{array}$$

where $\rho \left(p,q\right)$ represents the amplitude of the pixel after clutter suppression, and $DPCA_{C,m}\left(p,q\right)$, $DPCA_{T,m}\left(p,q\right)$, $DPCA_{N,m}\left(p,q\right)$, respectively, represent the complex data of the stationary ground clutter, the moving target, and the complex Gaussian noise signal processed by DPCA. Hypothesis $H_{0}$ means that a moving target is absent, and hypothesis $H_{1}$ assumes that a moving target exists.

**Figure 5:**
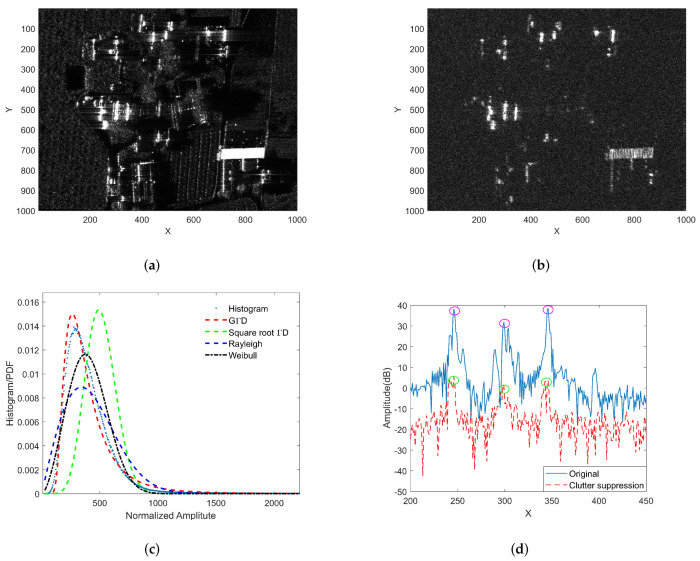
Clutter suppression and the PDF fitted by step II. (**a**) X-band 0.3 m resolution SAR image; (**b**) clutter suppression result; (**c**) PDF fitting result; (**d**) amplitude before and after clutter suppression: red and green ellipses indicate the amplitude of strong clutter before and after clutter suppression, respectively.

The next step is to select the distribution model and estimate the parameters from the image data. Every statistical distribution has its own applicable scenarios. In order to describe clutter precisely, the distribution model and parameter estimation method should be selected according to the specific application. Take the 0.3 m X-band SAR image as an example to study the amplitude distribution of the high-resolution SAR image after clutter suppression. The observation scene in Figure 5a is rural areas, including houses, farmland, trees, and other clutter, which can be considered as a heterogeneous clutter environment. Figure 5c shows the results of histogram-fitting of the data in Figure 5b through four probability distributions, which are generalized gamma distribution (GΓD), Weibull distribution, gamma distribution, the square root of gamma distribution, and Rayleigh distribution, respectively. It can be seen from the figure that the fitting effect of GΓD distribution is the best. For a heterogeneous clutter environment and high-resolution SAR image, the amplitude after clutter suppression no longer obeys the square root of gamma distribution but follows a heavy-tailed distribution [36,45,46]. Therefore, the GΓD distribution is used to characterize the background clutter in this paper. It is used for homogenous or inhomogeneous SAR-amplitude intensity image with multiple types of terrain, such as urban areas, farmland, and mountains.

The probability density function (PDF) of GΓD is written by [45]:

(24) $$f\left(x\right)=\frac{\left|v\right|k^{k}}{\sigma \Gamma \left(k\right)}{\left(\frac{x}{\sigma }\right)}^{kv-1}exp\left(-k{\left(\frac{x}{\sigma }\right)}^{v}\right)\;,\delta ,\left|v\right|,k,x>0$$

where $\Gamma \left(k\right)$ denotes the gamma function, and *k*, σ, *v* represent the shape parameters, power, and scale, respectively.

The key step prior to CFAR is how to accurately estimate the distribution parameters and calculate the threshold. Parameter estimation methods mainly include method of moments (MoM), the maximum-likelihood method, and the method of log-cumulants (MoLC). Compared to the other two methods, MoLC has higher parameter estimation accuracy. The relationship between the first three log-cumulants of GΓD and parameters *k*, σ, and *v* is written by [46]:

(25) $$\begin{array}{c} k_{1}=\ln\sigma +\frac{\left(\Psi \left(k\right)-ln\;k\right)}{v}\\ k_{2}=\frac{\Psi \left(1,k\right)}{v^{2}}\\ k_{3}=\frac{\Psi \left(1,k\right)}{v^{3}} \end{array}$$

where $\Psi \left(x\right)$, $\Psi \left(n,x\right)$, respectively, represent the digamma function and n-order Polygamma function, and $k_{1},k_{2},k_{3}$ are the first three log-cumulants of GΓD (its calculation method is given in [46]). The adaptive detection threshold *T* can be calculated using local background clutter around the pixels to be detected in the sliding window. Let $F\left(x\right)$ be the corresponding cumulative distribution function (CDF). The PFA can be given by [36,46]:

(26) $$P_{\mathrm{fa}}=1-\int_{-\infty }^{T}f\left(x\right)dx=1-F\left(x\right).$$

The incomplete gamma function is represented by $Q\left(x,k\right)$. Taking Equation (24) into Equation (26), we can obtain the CDF of the background clutter as by [45]:

(27) Fx=Qkxxσσv,k,v>01−Qkxxσσv,k,v<0.

By submitting Equation (27) to Equation (26), we can obtain the adaptive detection threshold *T* as by [45,46]:

(28) $$T=\left\{\begin{array}{c} \sigma {\left\{\frac{1}{k}Q_{inv}\left(1-P_{fa},k\right)\right\}}^{\frac{1}{v}},v>0\\ \sigma {\left\{\frac{1}{k}Q_{inv}\left(P_{fa},k\right)\right\}}^{\frac{1}{v}},v<0 \end{array}\right.$$

where $Q_{inv}\left(x,k\right)$ represents the inverse function of $Q\left(x,k\right)$.

From the above analysis, we obtain PDF, CDF, and detection threshold calculation methods. Now we verify the effectiveness of the proposed method through actual SAR data. In order to judge the performance of fitting, Kullback–Leibler divergence (KL) is used to evaluate the difference between the two probability distributions of the statistical histogram and fitting result. A smaller value of KL means that the distribution is more suitable for the data.

In a SAR-GMTI system, the PFA expresses the probability of an event that a “non-target” pixel can be detected as a “target” pixel. We use the ratio of the number of detected "no target" pixels to the number of image pixels to represent the actual false alarm rate (Actual FAR). The actual probability of a false alarm can be estimated by [45,47]:

(29) $$\hat{P_{\mathrm{fa}}}=\frac{N_{D}-N_{\mathrm{true}}}{N_{a}N_{r}}$$

where $N_{a}$ and $N_{r}$ represent the number of pixels in the azimuth and range directions of the SAR image, respectively. $N_{D}$ represents the number of detected pixels, and $N_{\mathrm{true}}$ represents the number of pixels occupied by the real targets.

The KL value and Actual FARs of the four methods are listed in columns 2 and 3 of Table 1, respectively. It can be seen from the table that GΓD has the minimum KL value and Actual FAR, which means it is the most effective method to fit the background among the four methods. However, Figure 5d intuitively shows that the residual energy of strong clutter is still large after clutter suppression. After the clutter-suppression operation, the clutter-to-noise ratio (CNR) is still greater than 13 dB. This remaining clutter will produce a large number of false alarms. In Figure 5a,b,d, the X-axis represents the sampling point in the range direction, and the Y-axis represents the sampling point in the azimuth direction.

**Figure 6:**
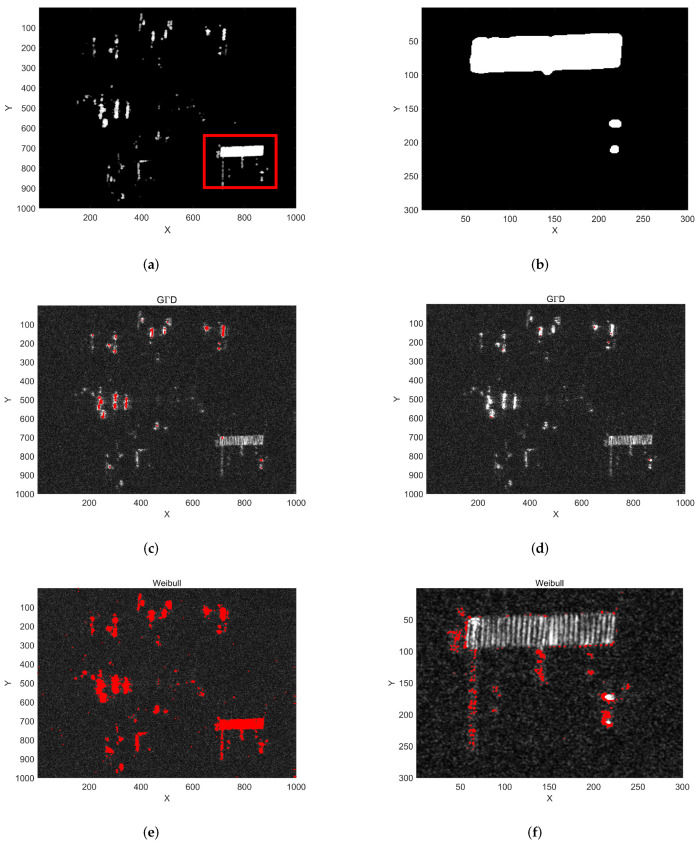
Detection results of the two methods: red pixels denote the position of false alarm. (**a**) Extracted strong clutter background; (**b**) enlarged view of red rectangular area in (**a**); (**c**) detection results of GΓD only by Step II; (**d**) detection results of GΓD Step I and Step II combined; (**e**) detection results of Weibull distribution only by Step II; (**f**) detection results of Weibull Step I and Step II combined.

The false alarm caused by strong clutter cannot be removed by traditional clutter suppression methods. Next, we combine the a priori strong clutter background obtained in Step I to further eliminate false alarm targets. The strong clutter background extraction result is shown in Figure 6a, and Figure 6b is an enlarged view of the red rectangular area in Figure 6a. Figure 6c–f only show the detection results of the two distributions (and the Weibull distribution) before and after combined Step I. The fourth column of Table 1 gives the Actual FARs of Step I and Step II combined. Comparing the processing results of the two methods, the clutter suppression method proposed in this paper can effectively reduce the number of false alarms caused by strong clutter.

### 3.3. Step III: A New Test Based on DLRVP

After the above two steps, most of the false alarm targets have been excluded, and the position of each potential moving target in the image has been obtained. However, there are still some isolated strong scattering points or edges of man-made objects that cannot be eliminated by the above two steps, as shown in Figure 6d,f. In order to solve this problem and realize radial velocity estimation, a new detection method based on interference phase after clutter suppression is proposed. The interferometric phase caused by the radial velocity of the moving target has a linear relationship between different channels, but there is no such relationship for static clutter. Therefore, we can use the above characteristic of multi-baseline interferometric phase to distinguish moving targets from stationary clutter.

Due to the size and defocus of the target itself, each moving target occupies multiple pixels in the high-resolution SAR image. Based on the above analysis, this paper puts forward two hypotheses. Firstly, it is assumed that the moving target is rigid and the speed of each part is consistent. Second, it is assumed that the target only runs forward without rotation [48,49,50] in the synthetic aperture time (usually about ten seconds for airborne SAR). These two assumptions are suitable for most ground-moving targets, such as passenger cars, trucks, etc. Therefore, the radial velocities of each component of a moving target can be considered to be equal. Assuming that the moving target contains K pixels in the SAR image, for heterogeneous clutter, the product model of $k_{th}$ pixel of M channels is given by:

(30) $$\begin{array}{c} Z\left(k\right)=\left[\begin{array}{c} \begin{array}{c} z_{1}\left(k\right)\\ z_{2}\left(k\right)\\ \vdots \end{array}\\ z_{M}\left(k\right) \end{array}\right]=\mu \left(k\right)+\Delta \left(k\right)\cdot C\left(k\right)+N\left(k\right)\in C^{M\times 1}\\ \mu \left(k\right)=a\cdot d\left(\vartheta \right)=\alpha e^{j\varphi }\left[\begin{array}{c} \begin{array}{c} 1\\ \exp\left(j\vartheta \right)\\ \vdots \end{array}\\ \exp\left(j\left(M-1\right)\vartheta \right) \end{array}\right] \end{array}$$

where $\vartheta =2\pi dv_{tr}/\lambda r\omega$ denotes the interferometric phase caused by the radial velocity of the moving target as shown in (16), and $d\left(\vartheta \right)$ represents the spatial steering vector of a moving target. The complex amplitude *a* is described as the reflectivity of the moving target, while Δ represents the texture feature describing the fluctuation of the clutter.

For heterogeneous clutter, the PDF of clutter texture Δ is given by [38]:

(31) $$f_{\Delta }\left(\sigma \right)=\frac{2{\left(v-1\right)}^{v}}{\Gamma \left(v\right)}{\left(\sigma \right)}^{-\left(2v+1\right)}exp\left(-\frac{v-1}{\sigma^{2}}\right)$$

where $\Gamma \left(v\right)$ denotes the gamma function, and *v* represents a shape parameter that describes the degree of clutter unevenness [51] (the larger the value of *v*, the more even the terrain).

This section takes the interferometric phase after clutter suppression as the research focus. In this paper, the difference between adjacent channels is used to suppress clutter. After DPCA, we can obtain M-2 interferometric phase diagrams. The interferometric phase of the $k_{th}$ pixel of the $m_{th}$ interferometric diagram is obtained by the following equation:

(32) $$\begin{array}{c} X\left(k\right)=\left[\begin{array}{c} \begin{array}{c} z_{2}\left(k\right)-z_{1}\left(k\right)\\ z_{3}\left(k\right)-z_{2}\left(k\right)\\ \vdots \end{array}\\ z_{M}\left(k\right)-z_{M-1}\left(k\right) \end{array}\right]=\Delta \mu \left(k\right)+\Delta C\left(k\right)+n\left(k\right)\in C^{\left(M-1\right)\times 1}\\ \phi_{m}\left(k\right)=\arg\left\{X_{m+1}\left(k\right)\cdot X_{1}^{*}\left(k\right)\right\} \end{array}$$

where m=1,⋯,M−2, and $\phi_{m}\left(k\right)\approx m\vartheta$ when a moving target is present, and $\Delta \mu \left(k\right)$, $\Delta C\left(k\right)$ represent the residual signal of moving target and clutter after clutter suppression, respectively. Noise is expressed in $n\left(k\right)$.

The multivariate PDF of ${(X\left(1\right),\cdots ,X\left(K\right))}^{T}$ can be written by [33]:

(33) $$\begin{array}{c} f_{X\left(1\right),\cdots ,X\left(K\right)}\left(X\left(1\right),\cdots ,X\left(K\right)\right)\\ =\frac{{\left(v-1\right)}^{-2K}\Gamma \left(v+2K\right)}{\pi^{2K}\det{\left(R\right)}^{K}\Gamma \left(v\right)}{\left(1+\sum_{k=1}^{K}\frac{{\left(X\left(k\right)-\Delta \mu \left(k\right)\right)}^{*}R^{-1}\left(X\left(k\right)-\Delta \mu \left(k\right)\right)}{v-1}\right)}^{-\left(v+2\right)} \end{array}$$

where *R* represents the covariance matrix of clutter plus noise.

The first element of the inverse of the Fisher information matrix can be used as an estimate of the Cramér–Rao bound (CRB) of the ATI phase [14,33]:

(34) $${\left(J^{-1}\right)}_{11}=\frac{\left(v-1\right)}{2Kv\alpha^{2}}{\left(d_{\vartheta }^{*}R^{-1}d_{\vartheta }-\frac{{\left|d_{\vartheta }^{*}R^{-1}d_{T}\right|}^{2}}{d_{T}^{*}R^{-1}d_{T}}\right)}^{-1}$$

where α is the amplitude of the moving target after clutter suppression, and the vector $d_{\vartheta }$ is the first derivative of the spatial steering vector $d_{X}={\left(1,\cdots ,\exp\left(j\left(M-2\right)\vartheta \right)\right)}^{T}$. So the CRB of the multichannel interference phase can be written by [38]:

(35) $$\begin{array}{c} \sigma_{\vartheta }=\sqrt{{\left(J^{-1}\right)}_{11}}\\ \;=\sqrt{\frac{\left(v-1\right)}{2Kv\alpha^{2}}{\left(d_{\vartheta }^{*}R^{-1}d_{\vartheta }-\frac{{\left|d_{\vartheta }^{*}R^{-1}d_{T}\right|}^{2}}{d_{T}^{*}R^{-1}d_{T}}\right)}^{-1}}. \end{array}$$

When the moving target is absent, the PDF of $\phi_{m}$ can be written by [14]:

(36) $$\begin{array}{c} f_{\phi_{m}}\left(\phi \right)=\;\frac{\Gamma \left(n+1/2\right)\left(1-\rho_{m}^{2}\right)\rho_{m}\cos\left(\phi -\varphi_{m}\right)}{2\sqrt{\pi }\Gamma \left(n\right){\left(1-\rho_{m}^{2}\cos^{2}\left(\phi -\varphi_{m}\right)\right)}^{n+1/2}}+\frac{{\left(1-\rho_{m}^{2}\right)}^{n}}{2\pi }{}_{2}F_{1}\left(n,1;\frac{1}{2};\rho_{m}^{2}\cos^{2}\left(\phi -\varphi_{m}\right)\right) \end{array}\;$$

where $\rho_{m}$ and $\varphi_{m}$, respectively, represent the amplitude and phase of the complex correlation coefficient between $X_{1}$ and $X_{m+1}$.

**Figure 7:**
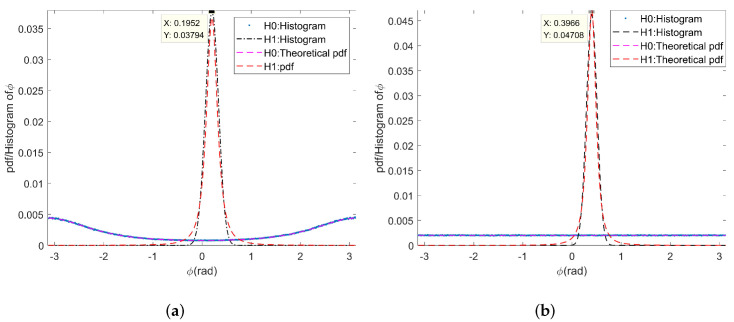
The theoretical PDF and histogram of interference phase: (**a**) $\phi_{1}$; (**b**) $\phi_{2}$.

Table 2 lists the simulation parameters of the four-channel SAR, and the theoretical PDF and statistical histogram of $\phi_{m}$ under $H_{0}$ and $H_{1}$ assumptions are shown in Figure 7. The simulation results verify that the phase corresponding to the peak of the PDF in Figure 7b is twice that in Figure 7a when there is a moving target.

As shown in (30) and Figure 7, the simulation results verify that the interference phase caused by the radial velocity of the moving target has a linear relationship between different channels, while the interferometric phase has no such relationship when only clutter exists. Therefore, we can use the above characteristic of the multi-baseline interferometric phase to distinguish a moving target from stationary clutter. Ideally, the interferometric phase of a moving target obtained from the M-2 interferograms after clutter suppression can be expressed by:

(37) $$\begin{array}{c} \Phi_{R}={\left[\phi_{1,1},\cdots ,\phi_{1,M-2};\phi_{2,1},\cdots ,\phi_{2,M-2};\phi_{K,1},\cdots ,\phi_{K,M-2}\right]}^{T}\\ \;\;\;=\vartheta \cdot {\left[1,\cdots ,M-2;\cdots ;1,\cdots ,M-2\right]}^{T}\in {}^{\left(M-2\right)\times K}. \end{array}$$

Considering the phase-wrapping problem, we consider transforming $\phi_{R}$ into Euler domain:

(38) $$\;\;\;\Phi \;\;{\;}_{R}^{EU}=e^{j{\;\;\;\Phi \;\;\;}_{R}}\in C^{\left(M-1\right)\times K}$$

Ideally, it is assumed that the scattering characteristics of K pixels occupied by moving targets are consistent and image registration, channel equalization, and interferometric phase correction are completed. Based on the above analysis, a new moving-target test variable based on multi-baseline interferometric phase is defined as (39):

(39) $$\beta =\left|\frac{\Delta {\left(\phi_{R}^{EU}\right)}^{H}\gamma \left(\vartheta \right)}{\sqrt{\Delta {\left(\phi_{R}^{EU}\right)}^{H}\phi_{R}^{EU}\Delta^{H}}\sqrt{\gamma {\left(\vartheta \right)}^{H}\gamma \left(\vartheta \right)}}\right|.$$

The element values of $\Delta \in R^{1\times K}$ are equal under homogeneous conditions, i.e., $\Delta =\left[1,\cdots ,1\right]$. The detection test represents the consistency between the observation matrix $\phi_{R}^{EU}$ and the spatial steering vector $\;\gamma \left(\vartheta \right)={\left[\exp\left(j\vartheta \right),\cdots ,\exp\left(j\left(M-2\right)\vartheta \right)\right]}^{T}\in C^{\left(M-2\right)\times 1}$.

Based on the above analysis, we can define a new detection method with the following hypotheses:

(40) $$\hat{\beta }\overset{H_{0}}{\underset{H_{1}}{≶}}\eta$$

where η denotes the detection threshold. When $\hat{\beta }$ is greater than η, it is considered that there is a moving target; while $\hat{\beta }$ is less than η, there is no moving target. In practice, it is difficult to obtain analytical expressions of false alarm probability and detection threshold. Thus, the detection threshold is determined by histogram fitting and a look-up table.

The maximum likelihood estimation of the ATI phase and radial velocity are given by:

(41) $${\hat{\vartheta }}_{ML}=\max_{\vartheta }\left|\frac{\Delta {\left(\phi_{R}^{EU}\right)}^{H}\gamma \left(\vartheta \right)}{\sqrt{\Delta {\left(\phi_{R}^{EU}\right)}^{H}\phi_{R}^{EU}\Delta^{H}}\sqrt{\gamma {\left(\vartheta \right)}^{H}\gamma \left(\vartheta \right)}}\right|$$

(42) $${\hat{v}}_{tr}=\frac{\lambda r\omega }{2\pi d}{\hat{\vartheta }}_{ML}.$$

The new detection method not only considers the velocity consistency of moving targets, but also makes use of the linear characteristics of multichannel interferometric phase of moving targets. When the moving targets are present, the interferometric phase of a moving target satisfies the linear relationship—the value of β is close to one. On the contrary, when only clutter exists, the interferometric phase no longer satisfies the linear relationship, the value of β is much smaller than one.

However, in practice the clutter is extremely heterogeneous, which means that the elements of vector Δ are not all equal. System error, non-stationary clutter, low signal-to-noise ratio, and other factors weaken the linearity of the interference phase between different channels, which will lead to the amplitude of β deviate from one. Therefore, it is necessary to analyze the peak position and the numerical distribution of β.

Firstly, the peak position of β is analyzed. Let $\Delta \phi_{m}=\phi_{m}-m\phi_{0}$ (in fact $\Delta \phi_{m}$ fluctuates near zero); for K→∞, the estimated value of β can be rewritten as:

(43) $$\begin{array}{c} \hat{\beta }=\frac{1}{M-2}\left|\sum_{m=1}^{M-2}E\left\{e^{j\Delta \phi_{m}}\right\}\right|\\ =\frac{1}{M-2}\left|\sum_{m=1}^{M-2}E\left\{\cos\left(\Delta \phi_{m}\right)+j\sin\left(\Delta \phi_{m}\right)\right\}\right|\\ =\frac{1}{M-2}\left|\sum_{m=1}^{M-2}E\left\{\cos\left(\Delta \phi_{m}\right)\right\}\right|\\ \approx \frac{1}{M-2}\left|\sum_{m=1}^{M-2}\cos\left\{E\left(\sqrt{\Delta \phi_{m}^{2}}\right)\right\}\right| \end{array}$$

where $E\left(\sqrt{\Delta \phi_{m}^{2}}\right)$ is the standard deviation of $\phi_{m}$. The analytical expression of $\hat{\beta }$ is not easy to obtain. Therefore, we use the CRB of ϑ to estimate the value of $\hat{\beta }$. When the target exists, the peak position of β is approximately located at:

(44) $$\hat{\beta }=\cos\left(\sigma_{\vartheta }\right).$$

From Equation (35), we can see that the main factors affecting the performance of $\hat{\beta }$ for a moving target are the number of looks K, the output SCNR, and the shape parameter of clutter. Next, the variation of peak position and detection probability with the above parameters is analyzed. Then, the influence of the above factors on the proposed test method is verified by Monte Carlo simulation. Finally, by comparing with the existing methods (such as traditional ATI, DPCA, and DRVC from the literature [14]), the effectiveness of this method for moving-target detection is illustrated. The following simulation is for non-stationary clutter, and the shape parameter of clutter is uniformly set to 3.1.

**Figure 8:**
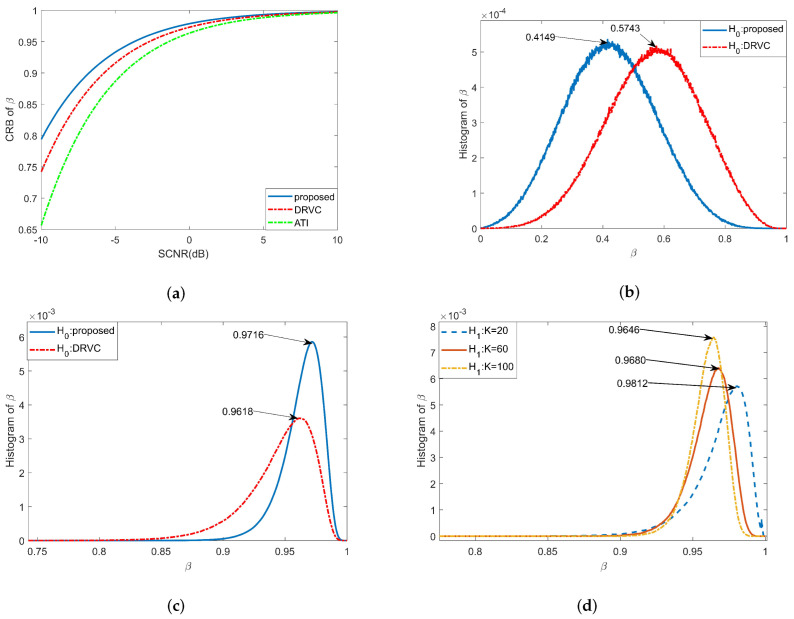
Simulation analysis of peak position of $\hat{\beta }$: $v_{tr}$ = 3 m/s, CNR = 13 dB. (**a**) The CRB of $\hat{\beta }$ with different input SCNR; (**b**) The histogram of $\hat{\beta }$ under hypothesis $H_{0}$; (**c**) The histogram of $\hat{\beta }$ under hypothesis $H_{1}$; (**d**) The histogram of $\hat{\beta }$ with different K.

**Figure 9:**
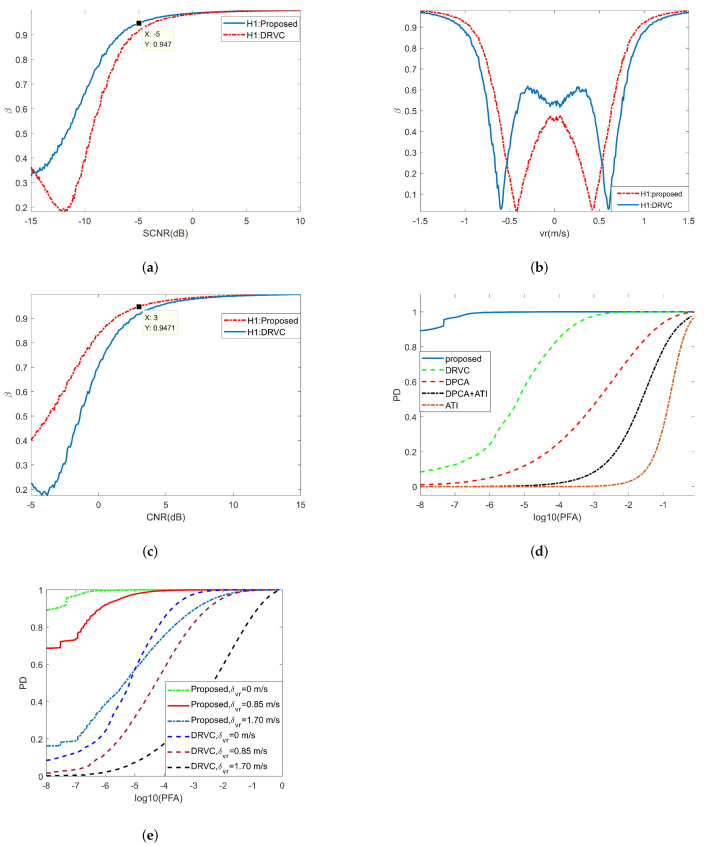
The performance analysis under hypothesis $H_{1}$. (**a**) $\hat{\beta }$ with different inputs for SCNR, $v_{tr}$ = 4 m/s, CNR = 13 dB; (**b**) $\hat{\beta }$ with different values for radial velocity of moving target, SCR = 10 dB; (**c**) $\hat{\beta }$ with different input for CNR, SCR = 5 dB; (**d**) ROC of five detection methods obtained by Monte Carlo simulations, SCR = 0 dB, $v_{tr}$ = 4 m/s; (**e**) ROC of two detection methods with different $\sigma_{vr}$.

The specific simulation results are analyzed as follows:

- 1. Peak position analysis of $\hat{\beta }$.
  The CRBs of $\hat{\beta }$ for three methods with different input SCNR are shown in Figure 8a. As can be seen from the figure, under the same simulation parameters, the peak value of the proposed method is the highest. Figure 8b shows the histogram of the new test and the DRVC test when the moving target is absent, and Figure 8c gives the histogram of above two methods when a moving target is present. When SCNR=0dB, the theoretical value of $\hat{\beta }$ is 0.9787, which is quite close to the simulation results in Figure 8c. From the simulation results, we prove that the proposed method can obtain greater $\hat{\beta }$ value under hypothesis $H_{1}$, which means that this method has better detection performance.
- 2. Main factors affecting $\hat{\beta }$.
  Figure 8d shows the histograms of the new test with different values for K (K = 20, 60, and 100). When the K value becomes larger, the peak position moves to the left and the width of PDF becomes narrower, which indicates better moving-target-detection performance. Figure 9a gives the Monte Carlo simulation results of $\hat{\beta }$ under different inputs for SCNR. When SCNR = −5 dB, the value of $\hat{\beta }$ has reached 0.9470, indicating that the new detection method can identify weak targets. Figure 9b shows the simulation results of $\hat{\beta }$ in the range of radial velocities of −1.5 m/s to 1.5 m/s. It can be seen that the new test has a narrower speed notch, which shows that the proposed method has better ability to detect slow targets; also, its minimum detectable speed is lower. The relationship between $\hat{\beta }$ and CNR is shown in Figure 9c. When CNR = 3 dB, the value of $\hat{\beta }$ has reached 0.9471, which means that the proposed method can adapt to a low-clutter environment (such as water surface and sea surface).
- 3. Detection performance of the new test.
  In order to prove the superiority of the proposed method, four existing detection methods (ATI, DPCA, DPCA+ATI, and DRVC) are compared and analyzed under the same simulation conditions. The ROC of the above methods obtained by Monte Carlo simulator is shown as Figure 9d. When the value of $P_{\mathrm{fa}}$ is as low as $10^{-7}$, the value of $P_{D}$ can still reach 0.9687. However, under this condition, the detection probability of DRVC is just 0.1261, and the detection probability of the other methods is lower. Under the same false-alarm probability, the proposed method can get the highest detection probability because it considers the linear characteristics of interferometric phase between channels.
- 4. Influence of radial velocity fluctuation on the new test.
  This paper assumes that the motion characteristics of each component of the moving target are consistent. However, in practical situations, factors such as terrain fluctuations, target rotation [48,49], and rapid maneuvers will weaken the consistency of target motion. Therefore, it is necessary to analyze the influence of radial velocity fluctuation on detection performance. The ROC of DRVC and the proposed test with different speed standard deviations $\sigma_{vr}$ are shown in Figure 9e. Under the same simulation conditions, with the increase of $\sigma_{vr}$, the detection probability of the proposed test and DRVC method decreases. However, the DRVC method has a larger decline, which shows that the proposed method has better speed robustness than the DRVC method and can better adapt to moving targets with certain speed fluctuations.

Compared to the other four methods, the advantages of the proposed method are summarized as follows:

- Peak position is larger and the false alarm probability is lower;
- Narrower speed notch with a smaller minimum detectable speed;
- Lower input SCNR requirements for moving targets;
- Higher detection probability than the four other methods;
- Stronger speed robustness and can better adapt to speed fluctuation;
- Can better suppress strong static isolated clutter.

## 4. Experimental Results

**Figure 10:**
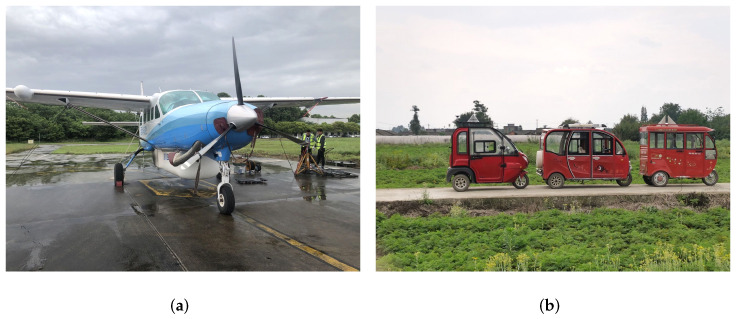
Flight platform and cooperative targets: (**a**) airborne SAR flight platform; (**b**) cooperative targets.

In this paper, four-channel airborne X-band data was used to verify the correctness and effectiveness of the proposed method. The radar working mode was one channel to transmit and four channels to receive simultaneously. The main parameters are shown in Table 3. The experimental platform is shown in Figure 10a. The experimental observation scene was a rural area with rich ground features (e.g., houses, roads, orchards, paddy fields, and other landforms with various clutter forms), which could better verify the adaptability of the method proposed. In the experiment, the three tricycles in Figure 10b were used as the cooperative target to verify the detection performance of the proposed method. Each tricycle was equipped with a corner reflector with an RCS of 1 m^2^ and a GPS device to measure the position and speed of the vehicle. In the experiment, the movement speed was controlled to be less than 3 m/s. The flight platform flew in a circle with a radius of 5 km in a clockwise direction. The BP algorithm was used to image the experimental data. The number of pixels in each image was 8000 × 8000. The pixel pitch was 0.1 m × 0.1 m, and the corresponding scene size was 800 m × 800 m.

### 4.1. Experiment A: Clutter Background Extraction

In the experiment, 44 continuous sub-aperture images were selected for research, corresponding to the aperture angle swept by the flying platform of approximately 36${}^{\circ }$. In order to ensure the correlation between adjacent sub-aperture images, only 2000 pulses were offset between two adjacent sub-aperture images during imaging, and the corresponding angular deviation was only 0.8${}^{\circ }$. Therefore, it can be considered that the scattering characteristics of the strong scatterer are approximately identical between adjacent sub-apertures.

**Figure 11:**
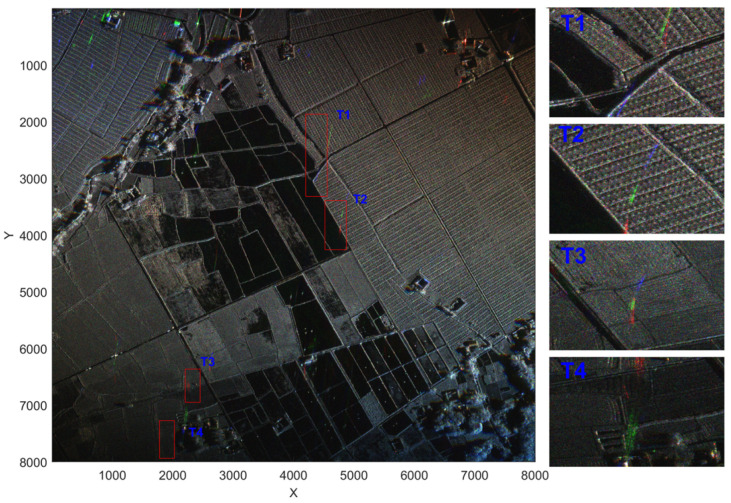
An RGB image synthesized from three sub-aperture SAR images with different angles; enlarged views of the moving-target area. The sub-aperture angles corresponding to R, G, and B are 0${}^{\circ }$, 18${}^{\circ }$, and 36${}^{\circ }$, respectively.

In order to reflect the difference between the position of the stationary target and the moving target in the image over time, we selected sub-aperture SAR images No. 1, No. 21, and No. 44 at three angles (about 0${}^{\circ }$, 18${}^{\circ }$, 36${}^{\circ }$) to synthesize RGB images, as shown on the left side of Figure 11. You can find four moving targets in the picture, three of which are cooperative targets, marked as T1, T2, and T3, and the other is a non-cooperative target, marked as T4.

**Figure 12:**
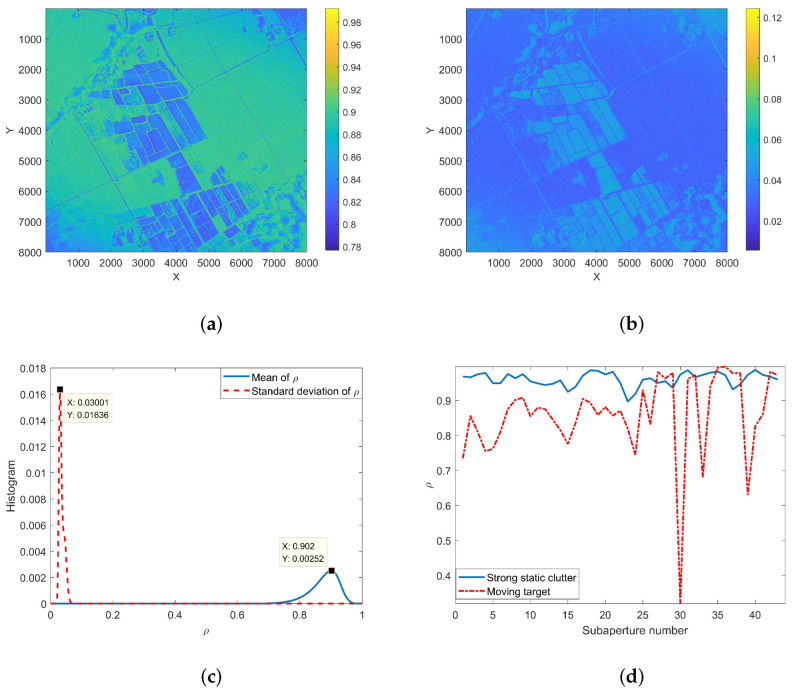
Correlation coefficient and its standard deviation. (**a**) Mean value of correlation coefficient; (**b**) standard deviation of correlation coefficient; (**c**) statistical histograms of correlation coefficient and its standard deviation; (**d**) correlation coefficients of the stationary target and the moving target vary with the aperture angle.

Figure 12a,b are the mean value and standard deviation of the correlation coefficient of 44 adjacent sub-aperture images in the time dimension, and the distribution histograms of the correlation coefficient and its standard deviation are shown in Figure 12c. It can be seen from the figure that the correlation coefficients for the position of the strong static clutter are relatively high, basically above 0.9.

**Figure 13:**
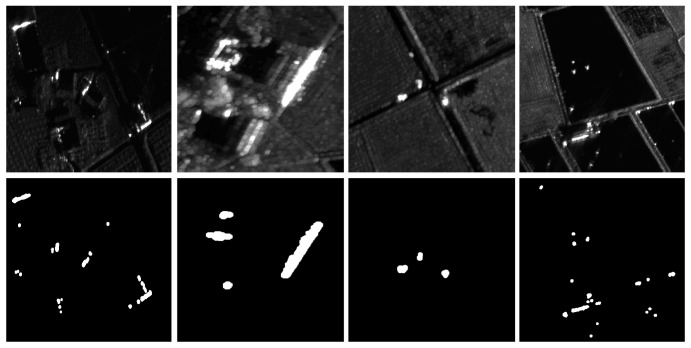
Strong clutter (**first row**) and extracted background (**second row**); from left to right: villages, houses, vehicles parked at intersections, and metal sprinklers in pools.

The imaging position of a moving target is different in different sub-apertures, so the motion trajectory is formed in the standard deviation image of the correlation coefficient. The distribution of the correlation coefficient of a single pixel in different sub-aperture images is shown in Figure 12d. In the experiment, the thresholds of the correlation coefficient and its standard deviation are set to 0.94 and 0.03, respectively. Figure 13 shows part of the strong clutter background and the corresponding background extraction results. From the figure, it can be seen that the proposed method can extract the contour and position information of the strong scatterer in the scene. The above experimental results prove the rationality of using the correlation coefficient between adjacent sub-apertures to eliminate strong clutter.

### 4.2. Experiment B: Clutter Suppression and Primary Detection

**Figure 14:**
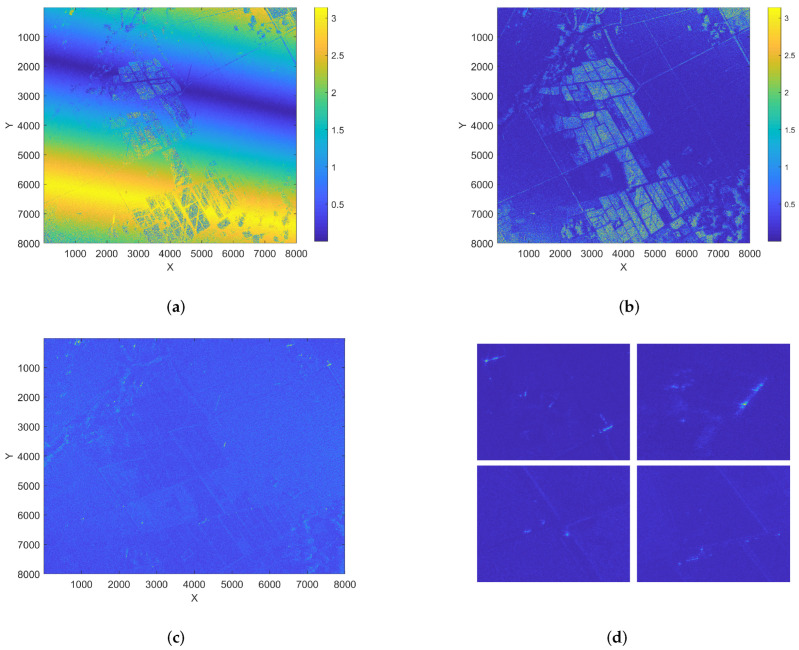
Interferometric phase and clutter suppression results. (**a**) Interferometric phase of channel 1 and channel 4 (before correction); (**b**) interferometric phase of channel 1 and channel 4 (after correction); (**c**) ${\mathrm{DPCA}}_{\max}$; (**d**) suppression results in strong clutter areas.

**Figure 15:**
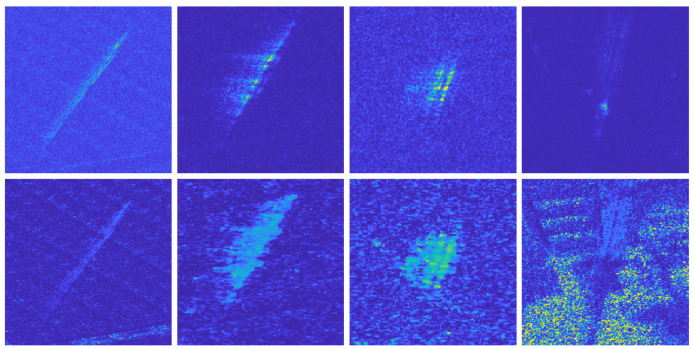
The clutter suppression results (**first row**) and interferometric phase (**second row**) of the target areas.

Next, we use the procedure in Section 3.2 to process the experimental data to achieve primary detection of moving targets in the scene area. Figure 14a,b show the interferometric phase of channel 1 and channel 4 before and after phase correction. It can be be observed in Figure 14a that there is a relatively obvious flat-ground phase before the phase correction. Then, using Equation (25) to suppress the clutter, the obtained clutter suppression result ${\mathrm{DPCA}}_{\max}$ is shown in Figure 14c. It can be seen from the figure that most of the clutter is effectively suppressed, but there is still a strong clutter residue in the strong clutter area, as shown in Figure 14d. If the moving target is detected directly in these areas, a large number of false alarms will be generated. The clutter suppression results and interferometric phase of the target area are shown in Figure 15.

**Figure 16:**
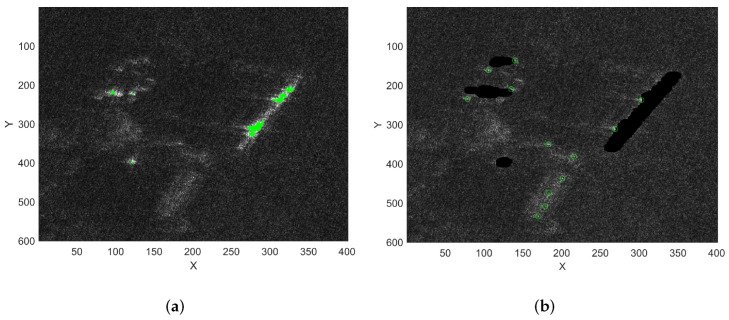
The detection results of Experiment B; green dots and green circles represent detected targets. (**a**) Only Step II; (**b**) Step I and Step II combined.

In the experiment, the false alarm probability is set as $P_{\mathrm{fa}}=10^{-7}$. Figure 16a shows the result of CFAR detection using only Step II, and Figure 16a shows the result of combining Step I and Step II for CFAR detection. Table 4 shows the detection threshold and the number of targets for the two processing methods. The experimental results prove that using the extracted clutter background as the prior information can effectively reduce the number of false alarms. At the same time, a lower detection threshold is obtained, which improves the detection ability of low-SCNR targets.

After the detection result of Figure 16a is clustered, there are still many false alarm targets, especially at the edges of strong scatterers or isolated scatter points. The intensity of these pixels is high, but the spatial similarity calculated by the method in Section 3.1 is low, which results in some pixels not being extracted as strong clutter background. In this case, even if Step I and Step II are combined to detect some pixels, false alarms will still be generated. Therefore, it is necessary to use the method in Section 3.3 to solve this problem.

### 4.3. Experiment C: The Proposed Detection Method and Radial Velocity Estimation

Based on the four-channel real data collected by the airborne SAR, we compare the detection performance of the proposed method with the DRVC method [14], the GO-DPCA method [11], and weighted DPCA [34].

Aiming at the problem that there are still many false alarms after Step I and Step II processing, this paper proposed a new moving-target-detection method. The proposed method uses the linear relationship between the interferometric phase generated by the radial velocity of the moving target and the space baseline to further reduce false alarms (see Section 3.3 for details). By setting different false alarm probabilities $P_{\mathrm{fa}}$, the number of pixels K occupied by the moving target in the image and whether or not the clutter background extracted by Step I is used, it was verified that the new detection method has more excellent detection performance and robustness than the existing DRVC and GO-DPCA methods and the weighted DPCA.

**Figure 17:**
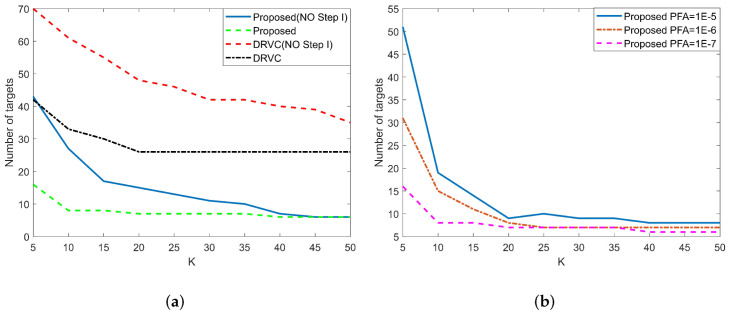
The number of detected targets for various values of K: (**a**) DRVC and proposed method; (**b**) under different $P_{\mathrm{fa}}$.

Figure 17a shows the change of the number of targets detected by DRVC and the method in this paper with the K value in the two cases of whether or not the clutter background extracted by Step I was used. Figure 17a shows the variation of the number of targets detected by the method in this paper with the K value under different $P_{\mathrm{fa}}$. Table 5 shows the number of targets and $\hat{P_{\mathrm{fa}}}$ (calculated by Equation (29)) detected by DRVC, GO-DPCA, the weighted DPCA and the proposed method when $P_{\mathrm{fa}}=10^{-7}$. It can be clearly seen from the comparison that the method proposed in this paper can effectively reduce the PFA. Compared to the DRVC method, when K = 5, the PFA of the proposed method is just 20% that of the DRVC method, and, at smaller values for K, the proposed algorithm can reach convergence.

**Figure 18:**
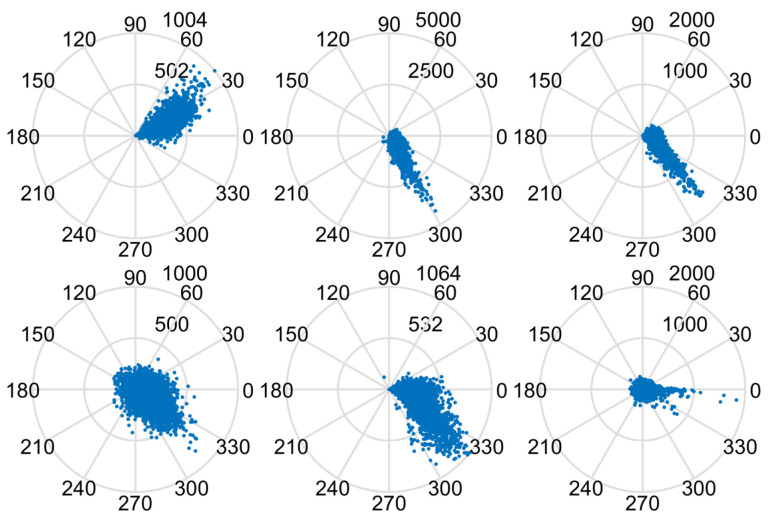
The amplitude and phase diagrams of the moving targets (T1∼T6).

Figure 18 shows the results of DRVC and the proposed method under the conditions of K = 40 and $P_{\mathrm{fa}}=10^{-7}$. The proposed algorithm detected six targets, among which T1∼T3 were cooperative targets and T4∼T6 were non-cooperative targets. In order to further confirm that there were no false-alarm targets in the detection results, we analyzed their amplitude and phase diagram. The interferometric phase between the first and fourth channels was taken as the observation phase, and ${\mathrm{DPCA}}_{\max}$ was the observation amplitude. A 300 × 150 data block around the target was intercepted to analyze the joint distribution of amplitude and phase. The amplitude and phase diagram of each target is shown in Figure 18. It can be seen from the figure that T1, T4, and T5 are severely defocused. The intensity of T6 is weaker and occupies fewer pixels. The interference phases of the six targets are 0.6865, −1.09, −0.8107,−0.7649,−0.6841, and −0.5248 rad, and the corresponding radial velocities are 0.8147, −1.1974,−0.9620,−0.81620, and −0.90727 m/s. The experimental results prove the proposed detection method can detect slow and weak targets with lower false-alarm probability and stronger robustness.

## 5. Conclusions

Aiming at the fact that the existing moving-target-detection methods cannot overcome the false-alarm problem caused by strong clutter residuals, a novel airborne CSAR moving-target-detection scheme is proposed. The scheme includes three steps: clutter background extraction, multichannel clutter suppression, and DLRVP detection. The important innovation of the proposed method is to use the linear relationship between the spatial baseline and the interferometric phase caused by the radial velocity of the moving target to eliminate the interference of strong clutter. In order to further prove that the proposed method has certain advantages compared to existing detection methods (DPCA, ATI, DPCA + ATI, DRVC, and weighted DPCA), the statistical distribution characteristics, peak position, detection probability, and resistance to speed fluctuations of the proposed model are simulated and analyzed. The measured data verify that the PFA of the proposed method is only 20% that of the comparison method and has better robustness.

## Figures and Tables

**Table 1:** KLs and Actual FARs, $P_{\mathrm{fa}}=10^{-5}$.

Distribution Function	KL	Actual FARs	
		Only Step II	Combined Step I and Step II
GΓD	0.03337	0.00106	0.00003
Rayleigh	0.41125	0.01131	0.00331
Weibull	1.62303	0.01812	0.00729
Square root GΓD	1.88569	0.01823	0.00738

**Table 2:** Simulation parameters.

Parameter	Value
Wavelength	0.032 m
Channel number	4
Adjacent channel spacing	0.1 m
Platform velocity	100 m/s
Velocity of moving target	1 m/s
CNR	13 dB
SCR	5 dB
Channel correlation coefficient	0.96
Clutter heterogeneity	3.1

**Table 3:** Main parameters of experimental data.

Parameter	Value
Band width	2000 MHz
Carrier frequency	10.0 GHz
Channel number	4
Pulse repletion frequency	2000 Hz
Velocity of moving target	<3 m/s
Adjacent channel spacing	0.095 m
Platform velocity	70 m/s
Flight radius	5 km

**Table 4:** Test results of Experiment B.

Method	Detection Threshold	Number of Targets
Step II Only	1762.5816	111
Step I and Step II Combined	553.9167	38

**Table 5:** Target detection results of three methods: $P_{\mathrm{fa}}=10^{-7};N_{a}=8000;N_{r}=8000;N_{\mathrm{true}}=6$.

Method		K									
		5	10	15	20	25	30	35	40	45	50
Proposed	$N_{D}$	16	8	8	7	7	7	6	6	6	6
	$\hat{P_{\mathrm{fa}}}/10^{-7}$	1.563	0.313	0.313	0.0156	0.0156	0.0156	0	0	0	0
DRVC	$N_{D}$	70	61	55	48	46	42	42	40	39	35
	$\hat{P_{\mathrm{fa}}}/10^{-7}$	10.000	8.594	7.656	6.563	6.250	5.625	5.625	5.313	5.156	3.125
GO-DPCA	$N_{D}$	140									
	$\hat{P_{\mathrm{fa}}}/10^{-7}$	20.938									
W-DPCA	$N_{D}$	163									
	$\hat{P_{\mathrm{fa}}}/10^{-7}$	24.531									

## Data Availability

Data sharing not applicable.
